# Abnormal TREC-Based Newborn Screening Test in a Premature Neonate with Massive Perivillous Fibrin Deposition of the Placenta

**DOI:** 10.1155/2016/5083274

**Published:** 2016-06-22

**Authors:** Stefan Kostadinov, Karen A. Robbins, Anthony Hayward

**Affiliations:** ^1^Department of Pathology and Laboratory Medicine, Women & Infants Hospital, 101 Dudley Street, Providence, RI 02905, USA; ^2^The Alpert Medical School of Brown University, 222 Richmond Street, Providence, RI 02903, USA; ^3^Division of Pediatric Allergy and Immunology, Hasbro Children's Hospital/Rhode Island Hospital, 593 Eddy Street, Providence, RI 02903, USA

## Abstract

Severe combined immunodeficiency (SCID), a primary immunodeficiency arising from variable defects in lymphocyte development and survival, is characterized by significant deficiency of thymus derived (T-) lymphocytes and variable defects in the B-lymphocyte population. Newborn screening for SCID is based on detection of low numbers of T-cell receptor excision circles (TRECs) by real time quantitative PCR (RT-qPCR). This screening allows for early identification of individuals with SCID and other disorders characterized by T-lymphopenia. Higher rates of abnormal screens are commonly seen in premature and critically ill neonates, often representing false positives. It is possible that many abnormal screens seen in these populations are result of conditions that are characterized by systemic inflammation or stress, possibly in the context of stress-induced thymic involution. We present a case of a male infant delivered at 27 weeks, 6 days of gestation, with severe intrauterine growth restriction who had an abnormal TREC screen and a* massive perivillous fibrin deposition* (MPFD) of the placenta. This association has not been reported previously. We are raising the awareness to the fact that conditions, such as MPFD, that can create adverse intrauterine environment are capable of causing severe stress-induced thymic involution of the fetus which can present with abnormal TREC results on newborn screening.

## 1. Introduction

Severe combined immunodeficiency (SCID), a primary immunodeficiency arising from variable defects in lymphocyte development and survival, is characterized by significant deficiency of thymus derived (T-) lymphocytes and variable defects in the B-lymphocyte population. Newborn screening for SCID is based on detection of low numbers of T-cell receptor excision circles (TRECs) by real time quantitative PCR (RT-qPCR) of dried blood spots, which are obtained for statewide newborn screening programs in the first days of life. TRECs are the products of T-cell receptor gene rearrangement and can serve as markers for output of naïve T-lymphocytes that have recently emigrated from the thymus. This screening allows for early identification of individuals with SCID and other disorders characterized by T-lymphopenia (e.g., 22q11.2 deletion syndrome/DiGeorge syndrome, ataxia telangiectasia), often before any clinical features are evident or recognized. However, increasing evidence and case reports suggest that other abnormalities may also be detected by low TREC counts [1].

Higher rates of abnormal screens have been observed in premature and critically ill neonates, often representing false positives, and many state protocols specifically include alternative screening algorithms to account for these findings [1]. While the explanation for these higher rates of low TREC counts in premature and critically ill neonates is not entirely evident, it may not always be explained by prematurity alone. It is possible that many abnormal screens seen in these populations are result of conditions that are characterized by systemic inflammation or stress, possibly in the context of thymic compromise or involution.

## 2. Case Report

Here we present a case of a male infant born at 27 weeks, 6 days of gestation, to a 20-year-old G1P0 mother. The prenatal history was significant for early onset intrauterine growth restriction and absent and subsequently reversed end diastolic flow as well as oligohydramnios. Prenatal labs and prenatal screening were within normal limits. Fetal growth restriction was first detected at 19 weeks and 4 days of ultrasound. At 26 weeks, 3 days, the fetus was below 2nd percentile with estimated fetal weight of 433 grams. The mother was admitted at 28 weeks for decreased fetal movements and fetal decelerations. Biophysical profile (BPP) was 4/10 with nonreactive Nonstress Test (NST). Emergency C-section was performed. Resuscitation was initiated with PPV for apnea, chest compressions for HR < 100 at 4 min, and intubation for apnea at 10 min. APGAR scores were 1/1, 3/5, 6/10, and 7/15 minutes, respectively. The infant's weight, length, and head circumference were below 3rd percentile. His 4-day course in the Neonatal Intensive Care Unit was significant for persistent suprasystemic pulmonary hypertension, worsening metabolic acidosis, anemia, and thrombocytopenia, and development of persistent right ventricular failure. On day of life 4 the infant developed acute decompensation and decision was made to withdraw care. The presumed cause of death was right heart failure secondary to pulmonary hypertension related to pulmonary hypoplasia.

Newborn screening was completed per standard operating procedure for the State of Rhode Island. Briefly, the first blood sample was collected between 24 and 48 hours of birth, on a standard newborn screening card, and sent to the New England Newborn Screening Lab (Jamaica Plan, MA). TREC analysis was completed by RT-qPCR, with use of RNAseP as an internal control. The result, 68, was outside the normal range (>270). Lymphocyte enumeration by flow cytometry was not able to be completed prior to death of the infant.

Autopsy consent was obtained. External measurements were consistent with severe growth restriction, with toe-heel length of 4.2 cm, corresponding to 23-24 weeks of gestation and femur length (taken from postmortem radiograph) of 3.5 cm, corresponding to 21-22 weeks of gestation. Postmortem examination was significant for pulmonary hypoplasia with lung/body weight ratio of 1.8% (<10th percentile for gestation) [2], right ventricular dilation, and evidence of right heart failure, including centrilobular congestion of the liver and congestion of the spleen and veins of other organs, including the testes. The lungs also demonstrated bilateral hyaline membrane formation and the liver demonstrated cholestatic changes. There was also evidence of significant perinatal stress, with severe thymic involution and moderate degree of normoblastosis. Notably, there was no evidence of significant inflammatory response within fetal tissues. Postmortem blood and lung cultures were negative for growth and karyotype analysis revealed a normal male chromosome complement.

**Figure 1:**
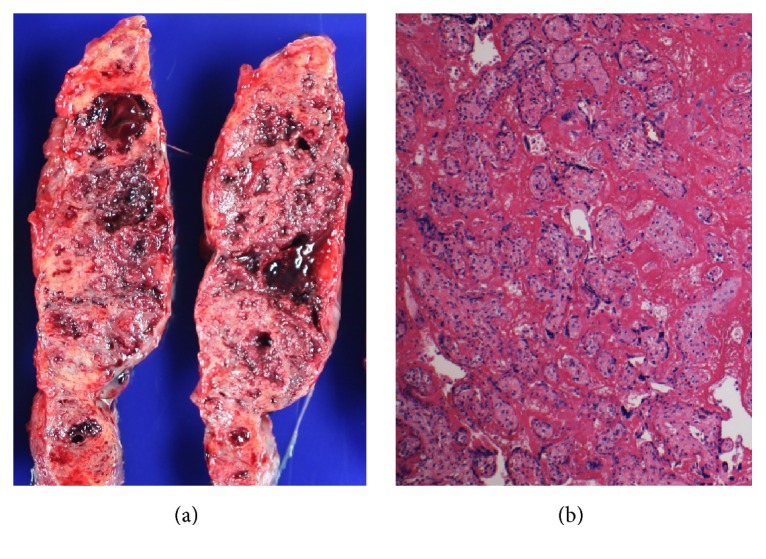
Placenta. Massive perivillous fibrin deposition. Cut surface of placenta with fibrous trabeculae forming a meshwork of confluent granular aggregates extending the entire thickness of the placental disc (a). Corresponding photomicrograph showing sclerotic chorionic villi encased with dense fibrin/fibrinoid material obliterating the intervillous space (b) (H&E, ×100).

Placental examination showed severe placental pathology dominated by* massive perivillous fibrin deposition* (MPFD) (Figure 1). There was near total (>80%) involvement of the placental parenchyma by perivillous fibrin and areas of villous infarction. In addition, a large subchorionic thrombohematoma was present, comprising approximately 45% of the fetal surface. Additional placental findings were multifocal villous edema, decidual vasculopathy, and moderate fetal normoblastemia. There was no evidence of significant maternal or fetal inflammatory response.

## 3. Discussion

Severe combined immunodeficiency (SCID) includes multiple genetic disorders that result in profound deficiencies in cellular and humoral immunity. SCID is often fatal if not recognized and treated within the first year of life [3]. The vast majority of infants with SCID appear physically normal at birth despite profound immunodeficiency with T-cell dysfunction. The asymptomatic clinical presentation together with the severe nature of the disorder, as well as the availability of effective treatment with hematopoietic stem cell transplant if performed at an early age, makes SCID an excellent condition for newborn screening. After initial pilot programs in Wisconsin and Massachusetts, several states have initiated universal, statewide newborn screening (NBS) for the early identification of SCID. The screen involves quantification of T-cell receptor excision circles (TRECs), which are nonreplicative pieces of DNA formed during T-cell receptor gene rearrangement in the thymus and serve as a useful biomarker for naïve T-cell lymphopoiesis. Screening has also identified other conditions characterized by T-cell lymphopenia, or secondary T-cell lymphopenia, in addition to SCID. Aggregated data analysis from 11 NBS screening programs in the United States showed that approximately a third of the non-SCID T-cell lymphopenias detected were syndromic conditions associated with T-cell impairment, DiGeorge syndrome/22q11.2 deletion, and trisomy 21 being most common. An additional 28% of the non-SCID cases were attributed to other medical conditions, the most common being congenital heart disease and other conditions associated with loss of lymphocytes into third space. Only 3% were classified as idiopathic or variant SCID where infants could not meet criteria for any other group but had persistent T-cell lymphopenia and immune dysfunction without defects in known SCID genes [1].

There have now been a number of publications reporting low TREC numbers among premature infants. Lymphocyte enumeration by flow cytometry can be performed as confirmatory testing for SCID but can be difficult to interpret in these infants due to the frequent administration of pre- and postnatal corticosteroids for complications related to prematurity and illness [4]. Furthermore, in cases such as ours described here, lymphocyte enumeration sometimes cannot be completed due to logistical reasons prior to death of the infant (delay in turn-around time after NBS sample obtained, limitations in ability to collect sample, etc.).

While there have been no known cases of SCID missed by NBS since implementation of the screening programs, there is a concern that the infants who died with inconclusive or abnormal TREC assays prior to obtaining a lymphocyte subset analysis may have died due to SCID or complications of T-cell lymphopenia [3]. However, in a retrospective chart review of 140, 533 infants screened for SCID/T-cell lymphopenia, 85% of those who died with abnormal or inconclusive TRECs were born before 33 weeks of gestation. The authors concluded that it was unlikely the infants died from complications of SCID or T-cell lymphopenia but rather from conditions related to prematurity and other common causes of death for this population [3].

Massive perivillous fibrin deposition (MPFD)/maternal floor infarction (MFI), two terms used interchangeably, is a placental pathologic condition with characteristic gross and microscopic features of excessive perivillous deposition of fibrinoid material. Although etiology is unclear, MPFD has a strong association with intrauterine growth restriction (IUGR), perinatal morbidity and mortality, and recurrence in subsequent pregnancies [5–7]. Deposition of perivillous fibrinoid material encases the chorionic villi and completely obliterates the maternal intervillous space, thus compromising the main functions of the placenta—exchange of nutrients and gases between fetal and maternal blood.

A classic triad of ultrasonographic findings of oligohydramnios; fetal intrauterine growth restriction (especially early onset); and a dense, hypoechogenic placenta have been noted as strongly suggestive of MPFD [6].

The causes of abnormal perivillous fibrinoid depositions appear to be associated with maternal alloimmune or autoimmune mechanisms as well as imbalances among factors that maintain the normal, fluid state of the blood in the intervillous space [8]. In addition, MPFD is characterized by imbalance of angiogenic/antiangiogenic factors in early pregnancy. Increased concentrations of soluble vascular endothelial growth factor receptor- (sVEGEFR-) 1 and soluble endoglin (sEng) have been detected in maternal plasma, both at the time of diagnosis and in the second trimester. A serial determination of these factors is proposed for monitoring future pregnancies at risk for MPFD. Treatment with statins (pravastatin) has been reported to reverse an antiangiogenic state and prevent fetal death in a mother with a history of four recurrent pregnancy losses and MPFD [9]. It has also been suggested that antibody-mediated maternal antifetal rejection plays a role in cases of MPFD [10].

Massive perivillous fibrin deposition in the placenta has been reported in association with various other maternal and fetal pathologies, such as Coxsackievirus [11], fetal renal tubular dysgenesis [12], maternal polymyositis [13], and mutations in long-chain 3-hydroxyacyl coenzyme, a dehydrogenase [14].

To our knowledge, MPFD has not been reported in association with abnormal TREC results or immunodeficiency states. Fetuses affected by MPFD of the placenta are experiencing prolonged intrauterine stress as evidenced by the significant morbidity and mortality characteristic for this condition. It has been shown that prolonged adverse intrauterine conditions result in stress-induced thymic involution of the fetus [15]. Multiple stress stimuli can act on the hypothalamic-pituitary-adrenal axis, leading to production of glucocorticoids. These hormones directly trigger apoptosis of double positive (DP) thymocytes. Additional involution pathways induced by stress stimuli can increase the production of IL-6, IL-10, and NGF and decrease growth hormone and dehydroepiandrosterone, in turn exacerbating thymic involution [16]. Therefore, it is plausible to assume that low numbers of TRECs on NBS reflect the decreased output of naïve T-lymphocytes from the stress-involuted thymus of these infants.

## 4. Conclusion

Maternal, fetal, or placental pathologic conditions that create adverse intrauterine environment can induce perinatal stress, especially in the premature infant. In such circumstances low values of TREC on newborn screening identify a potentially transient failure of T-cell production rather than SCID. This can cause undue anxiety among parents and generate confusion among clinicians. Identifying conditions, such as MPVD, that can explain the stress inducing adverse intrauterine environment can be important in the evaluation of inherited immune deficiency. Nevertheless, practitioners should be aware that premature infants with an abnormal TREC screen can still have true SCID or related immunopathology.
